# Auricular acupuncture for migraine: A systematic review protocol

**DOI:** 10.1097/MD.0000000000018900

**Published:** 2020-01-31

**Authors:** Feng Zhang, Yifeng Shen, Hongjuan Fu, Hao Zhou, Chao Wang

**Affiliations:** a Sichuan Integrative Medicine Hospital; b Acupuncture and Tuina School; c Clinical Medicine School, Chengdu University of Chinese Medicine, Chengdu, China.

**Keywords:** auricular acupuncture, migraine, protocol, randomized controlled trials, systematic review

## Abstract

**Background:**

Previous reviews indicate that the effect of auricular acupuncture on migraine. However, a systematic review is not available. Therefore, this protocol was conducted to evaluate the efficacy and safety of auricular acupuncture on migraine, by conducting a systematic review and meta-analysis.

**Methods:**

The following databases will be searched from their inception to October 2019: Chinese National Knowledge Infrastructure (CNKI), Chinese Biomedical Literature Database (CBM), Wan Fang Database, the Chongqing VIP Chinese Science and Technology Periodical Database (VIP), Cochrane Library, EMBASE, EBSCO, PubMed. The randomized controlled trials (RCTs) in English or Chinese associated with auricular acupuncture for migraines will be included. Eligible study conference abstracts and reference lists of manuscripts will be searched. The data collection and analysis will be conducted independently by 2 reviewers. Meta-analysis will be performed using Rev Man V.5.3.5 statistical software.

**Results:**

This systematic review will be conducted to evaluate the efficacy and safety of auricular acupuncture in the treatment of migraine. Therefore, auricular acupuncture in the treatment of migraine needs to be further clarified.

**Conclusions:**

In summary, this review will determine whether the impact of auricular acupuncture for intelligence on the treatment of migraines. A better approach may be established for migraine base on this review. It provides reliable evidence for its extensive application.

**Ethics and dissemination:**

The private information from individuals will not publish. This systematic review also will not involve endangering participant rights. Ethical approval is not available. The results may be published in a peer-reviewed journal or disseminated in relevant conferences.

**OSF REGISTRATION NUMBER:**

DOI 10.17605/OSF.IO/7ZR8Q

## 1 Introduction

The studies of epidemiology illustrated migraine, is a common disease with a 1-year prevalence of around 10% to 12% and a lifetime prevalence of between 15% and 20%.^[1]^ The prevalence of migraine is 14.9% in the United States, and 8.4% to 12.7% in Asia.^[2]^ The patients with migraine, approximately 25% to 38%, need therapy.^[3,4]^ Although pharmacotherapies (divalproex sodium, topiramate, metoprolol, and propranolol) are advised as a means for migraines. However, many patients experience adverse effects. Data from clinical trials show that dropout rates are high, suggesting that the drugs are not accepted by patients with migraine. In recent years, emerging evidence shows the influence of behavioral interventions on patients.^[5,6]^ But additional effective, low-risk treatments are desirable. It is a popular no pharmacological modality used for treating migraine.^[7]^ However, it is not simple and convenient to use requires sterile needles. Acupuncture was developed by a consensus of acupuncture experts. The patients need to remain in one position for long time during treatment.

In 2001, battlefield acupuncture was first developed by Niemtzow as an attempt to design an auricular acupuncture for reducing pain.^[9]^ The protocol of auricular acupuncture (cingulate gyrus, thalamus, omega-2, point zero, and Shen men) was stimulated sequentially in both ears. Auricular acupuncture is a traditional Chinese therapy according to a system of channels and meridians. It involves embedding therapy on-ear points, auricular acupoint seeds pressure, bloodletting at auricular points, auricular points on auto chemotherapy. Concerns were raised that auricular acupuncture is a simple, low-cost, and self-manageable nonpharmacological approach therapy for migraine.^[8]^ Previous reviews indicate that the effect of auricular acupuncture on migraine.^[9–13]^ Compared with acupuncture, it is a simple, safe, rapid, and effective modality in managing migraines.^[9–12]^ The auricular acupuncture interventions were simply developed by self after short training.^[14]^ However, there is a lack of evidence on the contribution of auricular acupuncture in the treatment of migraine. Therefore, this review will evaluate whether auricular acupuncture effective and safe compared with western medicine and acupuncture for the treatment of migraines. This review will be the first to evaluate the impact of auricular acupuncture.

## 2 Methods

### 2.1 Eligibility criteria

#### 2.1.1 Types of studies

We will eligible to study with randomized controlled trials (RCTs) using auricular acupuncture to treat migraines. Exclusion criteria included Quasi-RCTs.

#### 2.1.2 Types of participants

We included trials in which study participants had been diagnosed with migraines, according to the definition of the Headache Classification Subcommittee of the International Headache Society, people diagnosed with migraines. The study population initially included regardless of their age, race, or sex. Patients with the following conditions were excluded: patients have had a history of subarachnoid hemorrhage, cerebral hemorrhage, cerebral embolism, cerebral thrombosis, vascular malformation, arthritis, hypertension, arteriosclerosis, neurological diseases, immunodeficiency, and bleeding disorders or allergies.

#### 2.1.3 Types of intervention

##### 2.1.3.1 Experimental interventions

Auricular acupuncture (embedding therapy on-ear point, auricular acupoint seeds pressure, bloodletting at auricular points, auricular point on autohemotherapy) will be used in the Experimental interventions group.

##### 2.1.3.2 Control interventions

The control group will consist of western medicine or body acupuncture therapy. We excluded trials comparing auricular acupuncture and western medicine with western medicine.

#### 2.1.4 Types of outcome measures

##### 2.1.4.1 Primary outcome

For this study, the primary outcome is the overall effective rate.

##### 2.1.4.2 Secondary outcomes

The index of headache score and values of arteria cerebri anterior (ACA), vertebral artery (VA), middle cerebral artery (MCA) will be measured and evaluated using several instruments. Other outcomes included safety and adverse events of auricular acupuncture will be observed.

### 2.2 Search methods for identification of studies

The following databases will be searched from their inception to October 2019: Chinese National Knowledge Infrastructure (CNKI), Chinese Biomedical Literature Database (CBM), Wan Fang Database, the Chongqing VIP Chinese Science and Technology Periodical Database (VIP). Cochrane Library, EMBASE, EBSCO, PubMed. We will only include trials reported in English and Chinese. The search strategy for PubMed is shown in Table 1. Other electronic databases will be used the same strategy.

**Table 1:**
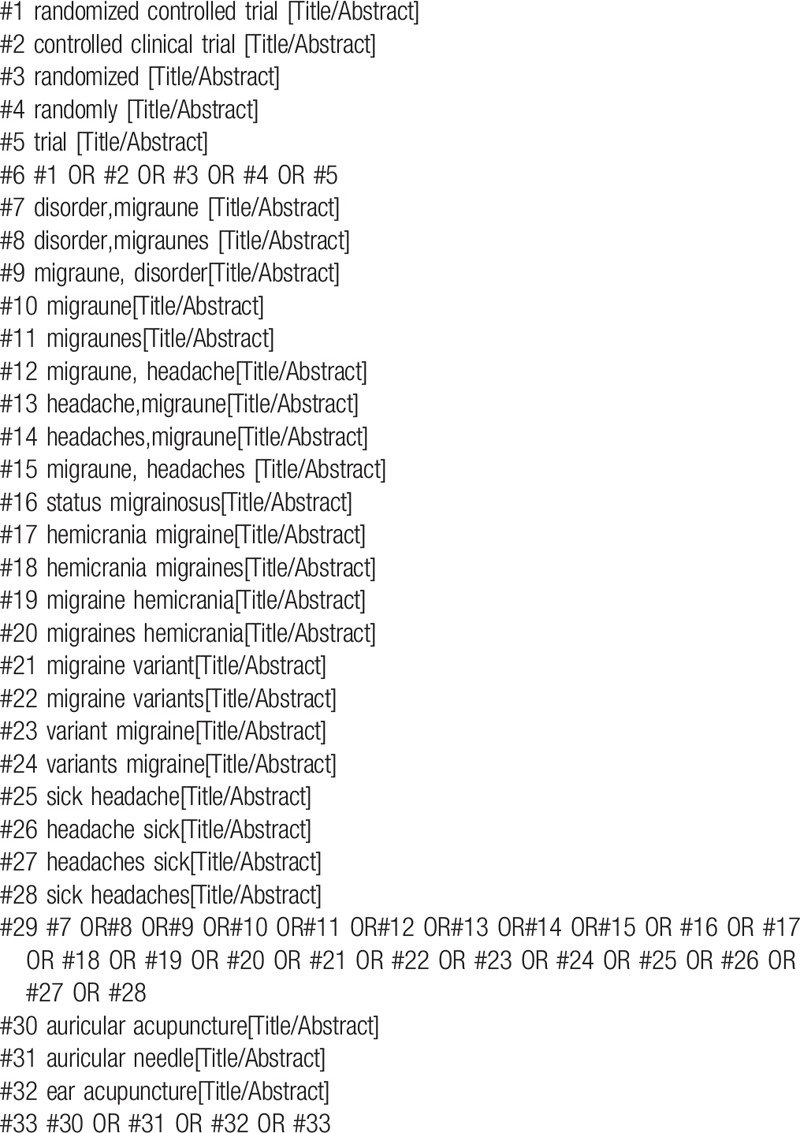
Search strategy in PubMed database.

### 2.3 Data collection and analysis

#### 2.3.1 Data extraction and management

**Figure 1:**
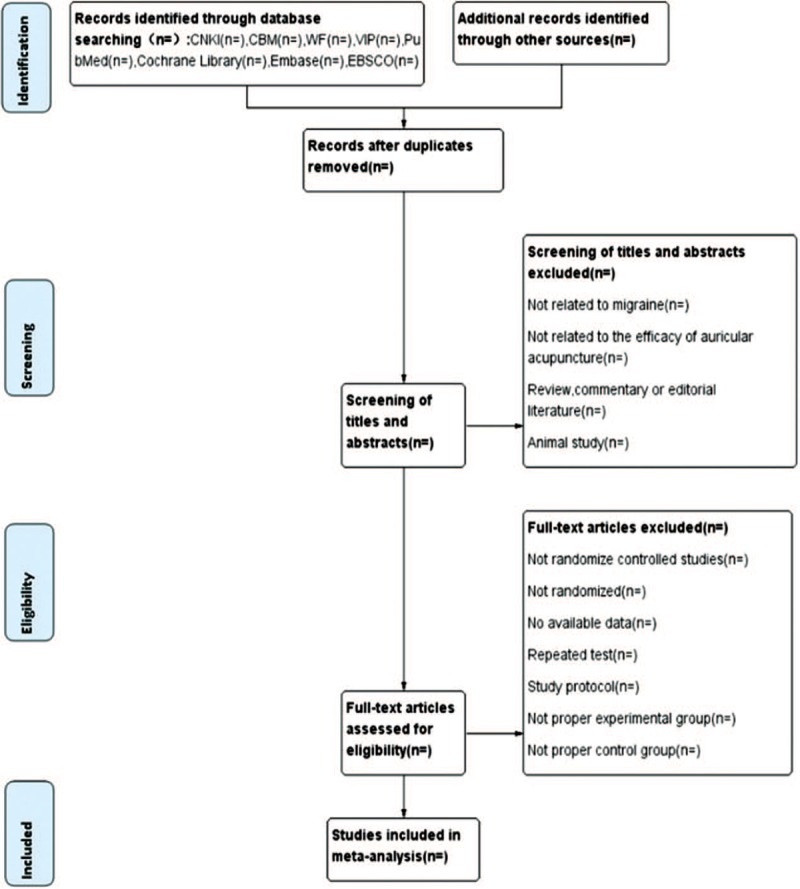
Flow diagram of study selection process.

The screening of studies will be conducted by 2 independent reviewers (ZF and FHJ). To assess whether the studies will be satisfied according to inclusion criteria after reading titles, abstracts, and full texts. Several studies from different opinions will be determined by the third reviewer (ZH). The study screening process is clarified for systematic reviews and meta-analyses flow chart (Fig. 1).

#### 2.3.2 Assessment of risk of bias

The assessment of risk of bias will be carried out by 2 independent reviewers (ZF and SYF), using the Cochrane Collaboration's “Risk of bias” tool. Study bias will be conducted as either: “unclear,” “low,” or “high” risk for the following criteria: random sequence generation, allocation concealment, blinding, incomplete data, selective outcome reporting, and other bias. The assessment of the bias has caused controversy, there is a need for discussion with a third reviewer (WC). The graphic representations of potential bias within and across studies using Rev Man V.5.3.5.

#### 2.3.3 Measures of treatment effect

Statistical analyses will be conducted using the risk ratio with 95% confidence intervals (CIs). Odds ratio (OR) and relative risk (RR) are commonly used for dichotomous outcomes data. For continuous outcomes, the weighted mean difference (WMD) or the standard mean difference (SMD) will be analyzed.

#### 2.3.4 Unit of analysis issues

The unit of analysis will be the individual participant.

#### 2.3.5 Dealing with missing data

Among the results of several studies with insufficient data or missing data, the corresponding author will be contacted to complement the contents. If the corresponding author cannot be contacted, the data alone will be conducted.

#### 2.3.6 Assessment of heterogeneity

The assessment of heterogeneity will be conducted by Review Manager (V.5.3.5). Chi-squared test and *I*^2^ value of the forest, plot will be calculated to assess heterogeneity, according to the Cochrane Handbook. The *I*^2^ value is classified into 4 levels: little or no heterogeneity (0%–40%), moderate heterogeneity (30%–60%), substantial heterogeneity (50%–90%), and considerable heterogeneity (75%–100%).

#### 2.3.7 Assessment of reporting biases

If the numbers of available studies are sufficient, funnel plots will be assessed reporting biases.

#### 2.3.8 Data synthesis

Review Manager (V.5.3.5) will be used to analyze. The test indicated little or no heterogeneity; a fixed effect model will be used for data. The random effect model will be adopted when there is considerable heterogeneity (*I*^2^ ≥ 50%). If there is considerable variation in results (*I*^2^ ≥ 75%), the meta-analysis will not be performed. The narrative and qualitative summary will be available.^[14]^

#### 2.3.9 Subgroup analysis and investigation of heterogeneity

Subgroup analysis will be conducted to assess heterogeneity. The different types of auricular acupuncture (embedding therapy on-ear point, auricular point seeds pressure, bloodletting at auricular points, auricular point on auto chemotherapy) may be affected heterogeneity.

#### 2.3.10 Sensitivity analysis

Sensitivity analysis will be used to assess the robustness of the results. It is possible to determine according to methodological quality, sample size, and analysis-related issues. The studies that follow a sequence will be removed from all the inclusion reviews. The chi-squared test and *I*^2^ value will be used to quantify statistical heterogeneity.

#### 2.3.11 Summary of evidence

The assessment of evidence for all outcomes will be summarized using the Grading of Recommendations Assessment, Development and Evaluation (GRADE) approach. The quality of evidence will be rated as high, moderate, low, and very low quality.

## 3 Discussion

Migraine is a disabling neurologic disorder. Despite this medical treatment, they are not necessarily effective for all patients, leading to serious adverse effects. It is no pharmacologic treatment options for migraine is required.^[14]^ Emerging evidence shows the influence of somatic acupuncture can be helpful in migraine treatment, but substantial data on auricular acupuncture are still lacking.^[15]^ Auricular acupuncture can be originated from ancient China. Studies are indicating the prevalence of use of auricular acupuncture among patients seeking migraine treatment.^[12,16–19]^ However, although several studies exist, the scientific mechanism of the effectiveness of auricular acupuncture is not completely available. Therefore, auricular acupuncture treatment in the treatment of pain needs to be further clarified.

This systematic review will evaluate published RCTs evidence for the effectiveness and safety of auricular acupuncture for acute migraine. This study has several strengths, it may assist clinicians and patient treatment for migraine with guidelines. Clinical research will be conducted based on this systematic review protocol. Acupuncture is appealing to some patients because the inconvenient is generally limited compared with auricular acupuncture. In summary, this review will be the first to evaluate the impact of auricular acupuncture for intelligence on the treatment of migraines. A better approach may be established for the treatment of migraine base on this review. It provides reliable evidence for its extensive application.

However, this systematic review also has several limitations. The treatment of auricular acupuncture includes embedding therapy on-ear point, auricular acupoint seeds pressure, bloodletting at auricular points, and auricular point on autohemotherapy. High heterogeneity may also arise from the various evolution standards from diffident ear acupuncture therapies. The studies published in English and Chinese will be searched. Other language studies were not available.

## Author contributions

**Data collection:** Yifeng Shen.

**Funding acquisition:** Chao Wang.

**Resources:** Hao Zhou.

**Software:** Hongjuan Fu.

**Supervision:** Chao Wang.

**Writing – original draft:** Feng Zhang.

**Writing – review and editing:** Hao Zhou.

## References

[1] Olesen J Lekander I Andlin-Sobocki P Funding of headache research in Europe. Cephalalgia 2007;27:995–9. 17727472 10.1111/j.1468-2982.2007.01397.x

[2] Wang SJ Epidemiology of migraine and other types of headache in Asia. Curr Neurol Neurosci Rep 2003;3:104–8. 12583837 10.1007/s11910-003-0060-7

[3] Pringsheim T Davenport W Mackie G Canadian Headache Society Prophylactic Guidelines Development Group. Canadian Headache Society guideline for migraine prophylaxis. Can J Neurol Sci 2012;39: suppl: S1–59. 22683887

[4] T felt-Hansen PC Evidence-based guideline update: pharmacologic treatment for episodic migraine prevention in adults: report of the Quality Standards subcommittee of the American Academy of Neurology and the American Headache Society. Neurology 2013;80:869–70. 10.1212/01.wnl.0000427909.23467.39 23439705

[5] Holroyd KA Penzien DB Pharmacological versus nonpharmacological prophylaxis of recurrent migraine headache: a meta-analytic review of clinical trials. Pain 1990;42:1–3. 2146583 10.1016/0304-3959(90)91085-W

[6] Nestoriuc Y Martin A Efficacy of biofeedback for migraine: a meta-analysis. Pain 2007;128:111–27. 17084028 10.1016/j.pain.2006.09.007

[7] Du R Wang Y Liu X Acupuncture for acute migraine attacks in adults: a systematic review protocol. BMJ Open 2015;5:e006968. 10.1136/bmjopen-2014-006968 PMC4401851 25869688

[8] Yeh CH Chien LC Huang LC Auricular point acupressure for chronic pain: a feasibility study of a 4-week treatment protocol. Holist Nurs Pract 2014;28:184–94. 24722613 10.1097/HNP.0000000000000027

[9] Niemtzow R Battlefield acupuncture. Med Acupunct 2007;19:225–8.

[10] Goertz CM Niemtzow R Burn SM Auricular acupuncture in the treatment of acute pain syndromes: a pilot study. Mil Med 2006;171:1010–4. 17076456 10.7205/milmed.171.10.1010

[11] Niemtzow R Belard JL Nogier R Battlefield acupuncture in the US military: a pain-reduction model for NATO. Med Acupunct 2015;27:344–8.

[12] Leggit JC Introduction of intergrative health and acupuncture to pre-clerkship medical students. Med Acupunct 2014;26:226–9. 25184015 10.1089/acu.2014.1045 PMC4142795

[13] Romoli M Allais G Airola G Benedetto C Ear acupuncture in the control of migraine pain: selecting the right acupoints by the ‘needle-contact test’. Neurol Sci 2005; 26(suppl):158–161. 10.1007/s10072-005-0434-5 15926019

[14] Gelfand AA Acupuncture for migraine prevention still reaching for convincing evidence. JAMA Intern Med 2017;177:516–7. 28241222 10.1001/jamainternmed.2016.9404

[15] Romoli M Allais G Airola G Ear acupuncture in the control of migraine pain: selecting the right acupoints by the “needle-contact test”. Neurol Sci 2005;26: suppl: S158–61. 15926019 10.1007/s10072-005-0434-5

[16] Lin YC Aimee B Lee A Acupuncture for the management of pediatric pain: a pilot study. Med Acupunct 2003;14:45–6.

[17] Allais G Romoli M Rolando S Ear acupuncture in the treatment of migraine attacks: a randomized trial on the efficacy of appropriate versus inappropriate acupoints. Neurol Sci 2011;32: suppl: S173–5. 21533739 10.1007/s10072-011-0525-4

[18] Allais G Romoli M Rolando S Ear acupuncture in unilateral migraine pain. Neurol Sci 2010;31: suppl: S185–7. 20464619 10.1007/s10072-010-0323-4

[19] Vincent CA A controlled trial of the treatment of migraine by acupuncture. Clin J Pain 1989;5:305–12. 2520420 10.1097/00002508-198912000-00006

